# A pragmatic randomized controlled trial of group transdiagnostic cognitive-behaviour therapy for anxiety disorders in primary care: study protocol

**DOI:** 10.1186/s12888-018-1898-1

**Published:** 2018-10-03

**Authors:** Pasquale Roberge, Martin D Provencher, Patrick Gosselin, Helen-Maria Vasiliadis, Isabelle Gaboury, Annie Benoit, Martin M Antony, Nils Chaillet, Janie Houle, Catherine Hudon, Peter J Norton

**Affiliations:** 1 Centre de recherche du Centre hospitalier universitaire de Sherbrooke (CRCHUS), Department of Family Medicine and Emergency Medicine, Faculty of Medicine and Health Sciences, Université de Sherbrooke, 3001, 12th Avenue North, Sherbrooke, QC J1H 5N4 Canada; 2 École de psychologie, Pavillon Félix-Antoine-Savard, 2325, rue des Bibliothèques, Université Laval, Québec, QC G1V 0A6 Canada; 3 Institut universitaire de première ligne en santé et services sociaux (CIUSSS de l’Estrie- CHUS), Department of Psychology, Université de Sherbrooke, 2500, boulevard de l’Université, Sherbrooke, QC J1K 2R1 Canada; 4 Department of Community Health Sciences, Université de Sherbrooke, Centre de recherche Hôpital Charles LeMoyne, 3120, boul. Taschereau, Greenfield Park, QC J4V 2H1 Canada; 5 Department of Psychology, Ryerson University, 350 Victoria Street, Toronto, ON M5B 2K3 Canada; 6 Department of Obstetrics, Gynecology, and Reproduction, Université Laval, 2705, boulevard Laurier, Québec, QC G1V 4G2 Canada; 7 Department of Psychology, Université du Québec à Montréal, C.P. 8888, succ. Centre-ville, Montréal, QC H3C 3P8 Canada; 8 Monash Institute of Cognitive and Clinical Neurosciences, School of Psychological Sciences, Monash University, 18 Innovation Walk, Clayton Campus, Clayton, VIC 3800 Australia

**Keywords:** Anxiety disorders, Cognitive behaviour therapy, Pragmatic trial, Group treatment, Primary care, Access to psychotherapy, Evidence-based practice, Psychotherapy, Cost/effectiveness, Transdiagnostic

## Abstract

**Background:**

Anxiety disorders are the most common mental disorders in community settings, and they are associated with significant psychological distress, functional and social impairment. While cognitive behaviour therapy (CBT) is the most consistently efficacious psychological treatment for anxiety disorders, barriers preclude widespread implementation of CBT in primary care. Transdiagnostic group CBT (tCBT) focuses on cognitive and behavioural processes and intervention strategies common to different anxiety disorders, and could be a promising alternative to conventional CBT. This study aims to examine the effectiveness of a transdiagnostic group CBT for anxiety disorders program as a complement to treatment-as-usual (TAU) in primary mental health care.

**Methods/Design:**

The trial is a multicentre pragmatic randomized controlled trial with a pre-treatment, post-treatment, and follow-up at 4, 8 and 12-months design. ***Treatment and control groups****.* a) tCBT (12 weekly 2-h group sessions following a manualized treatment protocol); b) TAU for anxiety disorders. Inclusion criteria comprise meeting DSM-5 criteria for primary Panic Disorder, Agoraphobia, Social Anxiety Disorder and/or Generalized Anxiety Disorder. Patients are recruited in three regions in the province of Quebec, Canada. The primary outcome measures are the self-reported Beck Anxiety Inventory and the clinician-administered Anxiety and Related Disorders Interview Schedule for DSM-5 (ADIS-5); secondary outcome measures include treatment responder status based on the ADIS-5, and self-reported instruments for specific anxiety and depression symptoms, quality of life, functioning, and service utilisation. ***Statistical analysis:*** Intention-to-treat analysis. A mixed effects regression model will be used to account for between- and within-subject variations in the analysis of the longitudinal effects of the intervention.

**Discussion:**

This rigorous evaluation of tCBT in the real world will provide invaluable information to decision makers, health care managers, clinicians and patients regarding the effectiveness of the intervention. Widespread implementation of tCBT protocols in primary care could lead to better effectiveness, efficiency, access and equity for the large number of patients suffering from anxiety disorders that are currently not obtaining evidence-based psychotherapy.

**Trial registration:**

ClinicalTrials.gov: NCT02811458.

## Background

### Background and rationale

Anxiety disorders are the most common class of mental disorders [1], with a past-year estimate prevalence of 11.6% [2]. They are characterized by excessive fear, anxiety and avoidance behaviour, often develop during youth and adolescence, and are frequently chronic in the absence of treatment [3]. Over half of cases are comorbid with other anxiety and depressive disorders [4–6], and comorbidity with other mental disorders [7] and chronic physical conditions is also common [8]. Considering that they are highly prevalent, disabling, comorbid, and with an early age of onset, anxiety disorders are accountable for an extensive personal, social and economic burden for the population [9–11]. Anxiety disorders are the sixth leading cause of years of life lived with disability [12]. The scaling-up of treatment for anxiety disorders could lead to substantial health benefits and economic return on investment [13]. As global health policies are increasingly oriented towards improving public mental health [14, 15], the strengthening of primary mental health care has mobilized attention as an approach to reduce the treatment gap for mental disorders. Primary care is generally the principal point of access to care for patients with mental disorders [16], and community-based mental health interventions could play a significant role in the improvement of the quality of care for anxiety disorders from a population health perspective.

Low rates of treatment adequacy have been found for anxiety disorders in studies conducted in various countries and clinical settings [16–22]. While both pharmacological and psychological treatments are recommended for anxiety disorders in clinical practice guidelines [23, 24], and most patients report a preference for psychotherapy [25], patients are increasingly treated with antidepressant medication [26]. A key issue in the treatment gap for anxiety disorders is access to evidence-based psychological interventions [22]. Cognitive Behaviour Therapy (CBT) is the most empirically supported psychotherapy for the treatment of anxiety disorders [27–29]. Among barriers to dissemination and uptake of CBT in routine care, psychologists and psychotherapists are scarce resources in primary care settings, with varying levels of training in evidence-based treatments [30]. In that context, providing access to a high-intensity individual psychotherapy with a therapist competent in CBT for specific anxiety disorders presents numerous challenges in community-based mental health care services. CBT can be delivered in various settings and formats [31–33], and treatment approaches that have a good potential for dissemination to clinicians and implementation in community settings are thus required to improve access to mental health care [34, 35].

Transdiagnostic CBT (tCBT) is a promising intervention to improve access to evidence-based psychological treatments for patients with anxiety disorders [34, 36–38]. By relating to a conceptualization of a general syndrome underlying the psychopathology of emotional disorders [37, 39, 40], tCBT offers an alternative to diagnosis-specific protocols by proposing evidence-based therapeutic procedures for a whole class of disorders, such as anxiety disorders [34, 41–43]**.** tCBT focuses on cognitive and behavioural processes that are shared among many disorders, as well as on common intervention strategies employed across the anxiety disorders (e.g., cognitive therapy, graduated exposure). tCBT encompasses an overarching transdiagnostic treatment protocol rather than multiple evidence-based psychological treatment protocols that target specific anxiety disorders, which could facilitate training and supervision of psychotherapists [34, 35]. tCBT could also be beneficial for patients who present a complex clinical profile as it allows simultaneous treatment of several comorbid pathologies for a given client rather than sequentially addressing multiple diagnoses with different treatments [44, 45]. A number of studies support the efficacy of individual, group and computerized tCBT for anxiety disorders [46–48]. From a primary mental health care standpoint, tCBT interventions in a group modality could be particularly valuable for the dissemination of CBT. Group tCBT for mixed-diagnosis anxiety disorders facilitates treatment planning and delivery, consequently allowing the clinicians to treat more patients per workday and reduce waitlist times in the context of limited resources and expertise [34]. tCBT group protocols relevant for multiple anxiety disorders have been developed by a number of research groups, and are generally associated with large effect sizes post-treatment [34, 47]. The tCBT group intervention for anxiety disorders developed by Norton [49] has demonstrated its efficacy through a number of trials conducted in a specialized university-based anxiety disorders research clinic [45, 50, 51].

Empirical data underscore the efficacy and feasibility of tCBT group approaches, which seem to be easily adaptable to the constraints of clinical settings and offer a promising solution to the challenges of comorbidity. Group tCBT should now be assessed in community-based primary mental health care, as transferability of the intervention from a specialized anxiety disorders clinic to community settings could contribute to improved access to evidence-based psychotherapy. In the context of the public mental health organization in the province of Quebec (Canada), community-based primary mental health care teams have been implemented in each administrative region since 2005 [52]. It is in their mandate to work in collaboration with family physicians to ensure access and continuity of care for their population, and they act as a gateway to specialized care. Clinical teams typically comprise psychologists, psychoeducators, social workers and nurses, and they are actively developing group treatments to meet high volume needs of the population for psychotherapy and manage their waiting lists, which is a good indicator of the feasibility and acceptability of group interventions for large-scale implementation.

In the present study, we aim to examine the effectiveness of group tCBT in the real world as it shows great potential to improve health outcomes for individuals with anxiety disorders. In the perspective of a pragmatic trial to examine the effectiveness of the intervention in terms of generalizability, feasibility and cost/effectiveness, we propose a comparative clinical effectiveness trial of group tCBT for anxiety disorders delivered in the primary care setting as a complement to usual care. Secondary objectives also encompass an economic evaluation as well as an embedded qualitative study to provide information to stakeholders on implementation issues.

### Objectives

The aim of the trial is to examine the effectiveness of group tCBT, as a complement to treatment-as-usual (TAU) in primary care, to reduce anxiety symptoms of adults with Panic Disorder, Agoraphobia, Social Anxiety Disorder and/or Generalized Anxiety Disorder compared to TAU. *Primary question*: When a group tCBT is added to TAU in primary care for a mixed group of patients with Panic Disorder, Agoraphobia, Social Anxiety Disorder and/or Generalized Anxiety Disorder, is it more effective to reduce anxiety symptoms than TAU alone? *Hypothesis*: Group tCBT will be more effective than TAU alone, showing the superiority of the tCBT intervention with usual care alone.

#### Secondary questions

a) Considering clinically significant outcomes for patients with anxiety disorders, is there a significant difference between groups in terms of remission rates and high-end functioning rates based on a clinician-rated measure? b) Is there a significant difference between groups on functioning and quality of life? c) Is there a differential treatment effect size based on diagnostic-specific measures for subgroups of patients meeting DSM-5 criteria for specific anxiety disorders at baseline? d) Is there maintenance of gains at follow up? e) Does the severity of depressive symptoms at baseline moderate treatment effectiveness for the primary outcome measures? f) Does the presence of an antidepressant and/or benzodiazepine medication at baseline moderate treatment effectiveness for the primary outcome measures? g) Is there differential treatment effectiveness based on the principal anxiety disorder at baseline? h) Is there a differential compliance rate and treatment effect size based on gender? i) Is tCBT as a complement to TAU preferable to TAU only based on a cost-effectiveness and cost-utility analysis (i.e., economic evaluation, not described in the protocol)? j) What are the acceptability and feasibility of the tCBT intervention for patients and therapists (i.e., embedded qualitative study, not described in the protocol)?

### Trial design

The trial is an investigator-initiated study designed as a two-arm parallel group multicentre pragmatic superiority RCT, with a 1:1 allocation at the level of the individual. The proposed trial conforms to the SPIRIT guidance for protocols of clinical trials [53].

## Methods

### Participants, interventions and outcomes

#### Study setting

The trial is being conducted in three health and social service administrative regions in the province of Québec, Canada. The network organization in Québec comprises regional integrated health and social services centres accountable for the delivery of care and services within a population responsibility framework. The universal health insurance system provides health care and social services coverage for the population. However, the organisation and delivery of mental health care varies considerably within each administrative region, and the same variability also applies to complementary mental health services available in the private sector (e.g., psychologists in private practice). We purposefully selected the following three study sites based on diversity (e.g., population size, region, university teaching hospital, psychiatric hospital, primary care mental health services, primary care network). The integrated health and social services centre for Quebec City and adjacent areas (i.e. *CIUSSS de la Capitale-Nationale)* serves an urban and rural population of 737,000 inhabitants over a large territory of 18,600 KM^2^; the study site is in the Quebec City region (89% of the territory population). The health and social services centre for the region of Laval (i.e. *CISSS de Laval*) serves a predominantly urban population of 435,000 inhabitants and is part of the Greater Montreal metropolitan area (over four million inhabitants). The health and social services centre for the Estrie region (i.e. *CIUSSS de l’Estrie - Centre Hospitalier universitaire de Sherbrooke)* serves a population of 474,000 inhabitants over a large territory of 13,000 km^2^; the study site is in Sherbrooke (> 164,000 inhabitants). All selected sites agreed to provide in-kind support for the study, including: 1- public sector psychologists and psychotherapists participation as co-therapists in treatment groups; 2- office space in primary care settings for participant’s assessment and treatment delivery. The institutional review boards at each site approved the protocol.

#### Eligibility criteria

In a pragmatic approach to eligibility and patient recruitment, the clinical trial is being conducted with a community sample of patients with anxiety disorders. Based on current knowledge about heterogeneous clinical profiles of patients with anxiety disorders in the primary care setting [22], eligibility criteria are as broad as feasible in terms of anxiety severity, psychiatric comorbidity, diagnosis status and ongoing treatments to reflect real life primary care patients. We include participants meeting the following inclusion criteria: (1) aged 18 to 65; (2) fluent in spoken and written French; (3) meeting DSM-5 diagnostic criteria for at least one of the following anxiety disorders as a primary mental disorder: Panic Disorder, Agoraphobia, Generalized Anxiety Disorder and/or Social Anxiety Disorder according to a clinical severity rating ≥ 4 for the Anxiety Disorders Interview Schedule for DSM-5 (ADIS-5) [54]. We exclude patients meeting the following exclusion criteria: (1) active suicidal intentions, psychosis, bipolar disorder, and active substance-related and addictive disorder in the past 12 months; (2) marked cognitive impairment [55]; (3) consultation with a psychiatrist in the past 12 months. These exclusion criteria were selected on the basis that it is unlikely that patients with this clinical profile would fully benefit from the group, and that patients accessing specialized mental health care would not be typical cases for primary care tCBT group treatment. Co-occurring mild to moderate psychiatric disorders, including major depression, obsessive-compulsive disorder or posttraumatic stress disorder, are permitted.

#### Interventions

##### Group tCBT for anxiety disorders plus treatment-as-usual (TAU)

The experimental condition is the transdiagnostic group cognitive-behaviour therapy (tCBT) program published by Norton (2012) [49]. The tCBT intervention encompasses four components: 1- *Education and self-monitoring:* education on the nature of anxiety and its treatment, and introduction to self-monitoring of triggers and responses to anxiety-provoking stimuli; 2- *Specific cognitive restructuring:* thinking errors and automatic thoughts associated with anxiety; 3- *Graduated exposure and response prevention:* gradual confrontation of fear-provoking stimuli; 4- *Generalized cognitive restructuring:* examining core beliefs and perceptions of everyday life. Table 1 presents the outline of the protocol. A written treatment protocol guides the delivery of the groups for the therapists. Patients receive a tCBT workbook at the first treatment session. The tCBT group includes weekly 2-h sessions with 8–10 patients during a 12-week period. A brief telephone or face-to-face individual preliminary contact is established with each participant by one of the co-therapists during the 2 weeks prior to the first session of tCBT to introduce the treatment and build an exposure hierarchy.

**Table 1:** tCBT treatment components

Components	Strategies
Psychoeducation (1.5 sessions)	Components of anxiety
	Treatment rationale
	Causes of anxiety
	Daily self-monitoring
Cognitive Restructuring (1.5 sessions)	Identify anxiety thoughts
	Identify misinterpretations and misappraisals
	Challenge and develop balanced interpretation or appraisal
Exposure (6 sessions)	Develop Fear Hierarchy
	Conduct in-session and homework exposure while engaging in response prevention
Schema-Based Cognitive Restructuring (2 sessions)	Identical to previous Cognitive Restructuring, but emphasis on general neurotic style
	The “tendency to interpret neutral or ambiguous stimuli as negative, threatening, and personally relevant”
Termination/Relapse Prevention (1 session)	Continued self-exposure and cognitive restructuring
	Lapses vs. relapses
	Emergency Action Plans

Groups are being delivered by two psychologists or psychotherapists accredited by the provincial regulatory body for the practice of psychotherapy. Primary therapists are PhD level private practice psychologists with a CBT approach and at least 2 years of clinical experience to ensure basic CBT competence for treatment fidelity. In a pragmatic perspective on treatment delivery, the co-therapists are selected by the health care managers at each study site (in-kind contribution) to reflect the range of clinical backgrounds of therapists working in the public sector in Québec. The training of co-therapists also supports capacity building and mentorship in tCBT in public sector mental health care services. Therapists’ training was initially provided during a centralized two-day workshop with the tCBT protocol developer (PJN), and followed by ongoing supervision by investigators (MDP, PG). In the event of the need to recruit more therapists after the initial training session, tailored individual training are provided to therapists (MDP). Supervision (MDP, PG) is provided to co-therapists at predefined times during the delivery of the intervention (pre-treatment, between sessions 3 and 4, between sessions 6 and 7, between sessions 10 and 11, post-treatment), as well as as-needed. Fidelity to the tCBT program is monitored through the recording of all treatment sessions. Therapeutic treatment adherence and competence is assessed by a random review of 30% of audio recordings during all sessions with a 5-point treatment integrity rating scale developed by the author of the intervention (PJN) [56] and used in previous trials [51, 57, 58]. Patient compliance is supported through high-quality interventions (e.g., trained and experienced therapists, supervision, monitoring treatment adherence and competence) and the provision of group interventions in a primary care setting (e.g., access, reduced mental health stigma) at convenient times (including evenings).

##### Treatment-as-usual

The TAU control group continues receiving usual care as a comparator. For this pragmatic clinical trial, no restrictions are imposed upon participation regarding having a family physician or treatment of mental disorders, as we are interested in comparing the added value of tCBT in real world conditions. Service utilization is being documented at each assessment period throughout the trial. As in other pragmatic trials, it should be noted that participation in the study may be associated with uncontrolled variations in mental health care seeking among patients for several reasons (e.g., instilling hope and motivation; awareness through comprehensive mental health assessment; knowledge about CBT; waiting for delayed intervention in the control group).

#### Participant assessment

A description of patient assessment timeline is presented in Table 2. They comprise instruments with good psychometric properties, and previously used in transdiagnostic and diagnostic-specific interventions for anxiety disorders to facilitate comparability [34, 59].

**Table 2:** Study schedule of patient assessment

Timepoint	Enrolment	Intervention	Follow-up			
	-T_1_		T_1_	T_2_	T_3_	T_4_
Clinician-administered measures						
Anxiety and Related Disorders Interview Schedule for DSM-5 (ADIS-5)	X		X		X	
Sociodemographic variables	X		X	X	X	X
Service utilization and medication	X		X	X	X	X
World Health Organization Health and Work Performance Questionnaire	X		X	X	X	X
Self-administered booklet						
Beck Anxiety Inventory	X		X	X	X	X
Social Phobia Inventory	X		X	X	X	X
Penn State Worry Questionnaire	X		X	X	X	X
Panic Disorder Severity Scale	X		X	X	X	X
Mobility Inventory for Agoraphobia	X		X	X	X	X
Patient Health Questionnaire	X		X	X	X	X
Insomnia Severity Index	X		X	X	X	X
Sheehan Disability Scale	X		X	X	X	X
EuroQol (ED-5D)	X		X	X	X	X
CDC Healthy Days Measures	X		X	X	X	X
Disease Burden Morbidity Assessment	X		X	X	X	X
Mental Health Self-Management Questionnaire	X		X	X	X	X
Mental Health Continuum –Short Form	X		X	X	X	X
MOS Social Support Survey	X		X	X	X	X
Completed during therapy (tCBT GROUP)						
Anxiety Disorder Diagnostic Questionnaire - weekly		X				
Credibility/Expectancy Questionnaire		X				
Working Alliance Inventory		X				
Gross Cohesion Scale		X				

##### Diagnostic interview

The **Anxiety and Related Disorders Interview Schedule for DSM-5** (ADIS-5) [54] is a semistructured interview designed to assess DSM-5 diagnostic criteria for anxiety disorders and comorbid mental disorders (e.g., mood disorders, substance use). The interviewer determines the presence of symptoms and rates their clinical severity (CSR) on a scale from 0 (no symptoms) to 8 (extremely severe symptoms) with increasing values indicating increased distress/interference, and a clinical threshold of 4 or higher. The ADIS-5 is initially used to assess eligibility criteria for the study. Co-occurring mild to moderate major depression, obsessive-compulsive disorder or posttraumatic stress disorder are permitted at the following conditions: a) CSR of 6 or lower; b) CSR at least one-point lower than for the principal anxiety disorder. The ADIS-5 will also be used to examine treatment outcomes through the dimensional CSR scales for each mental disorder. The clinician-rated measure of outcomes will be based on the CSR of the ADIS-5 [54] for the principal anxiety disorder. While there are no data on the psychometric properties of the ADIS-5, the ADIS-IV was shown to have good interrater reliability when assessing the presence of mental disorders [4]. Interrater reliability will be assessed for 25% of audio-recorded ADIS-5 diagnostic interviews at each assessment period by randomly selected clinical evaluators.

The initial interview also comprises a clinician-administered questionnaire on sociodemographic data, and a brief structured interview on health care costs used in previous studies [32] to obtain information on mental health consultations (e.g., type of professional, duration, costs), psychotropic medication (name, dosage, length of time), indirect costs (e.g., absenteeism) and other related variables such as previous therapy experience and treatment preference. The questionnaire also examines work performance based on the short version of the **World Health Organization Health and Work Performance Questionnaire** (HPQ) [60].

##### Primary outcome measures

Two primary outcome measures are included in the study to represent self-reported anxiety symptoms as well as clinician-rated assessment for the principal anxiety disorder diagnosis. As a self-reported generic measure of anxiety symptoms, we use the **Beck Anxiety Inventory** (BAI) [61, 62]. The BAI is a 21-item measure of emotional, physiological and cognitive symptoms of anxiety that can be used in primary care patients with different anxiety disorders. With scores ranging from 0 to 63, the interpretation of the scale corresponds to *minimal anxiety* (0–7), *mild anxiety* (8–15), *moderate anxiety* (16–25) and *severe anxiety* (26–63). Previous studies have established significant reliable improvement and clinically significant change cut-points for the BAI based on normative data and comparable samples of multiple anxiety disorders [63]. As a clinician-rated assessment, we will use the **CSR of the ADIS-5** for the principal anxiety disorder. Previous studies have used the ADIS-5 as a primary outcome measure for mixed anxiety disorders (e.g., [64]).

##### Secondary outcome measures

As secondary outcome measures with the ADIS-5, we will examine **high end-state functioning** as a post-treatment CSR of “2” or lower and **treatment responder status** as a post-treatment CSR < 4.

Participants also complete diagnostic-specific measures, quality of life and functioning measures, as well as other questionnaires related to anxiety disorders. The **Social Phobia Inventory** (SPIN) [65, 66] consists of 17 items measuring the fear, avoidance, and physiological discomfort commonly experienced by people with social anxiety disorder. Each item receives a rating from 0 to 4, with higher ratings indicating higher levels of distress associated with each statement. This questionnaire has good internal reliability, test-retest reliability, convergent validity, and a cut-of score of 19 can distinguish between patients with and without social anxiety with 79% accuracy [65]. The **Penn State Worry Questionnaire** (PSWQ) [67, 68] is a 16-item questionnaire to assess the trait of worry, which characterizes generalized anxiety disorder (GAD). Patients are asked to rate how well statements about worry describe themselves from 1 to 5. If the statement does not describe them at all, it is given a rating value of 1, whereas a statement that is very typical of them would receive a rating value of 5. It assesses the trait of worry with high internal consistency and test-retest reliability. The **Panic Disorder Severity Scale Self Report** (PDSS-SR) [69, 70] asks respondents to indicate the severity of each of 7 dimensions of panic disorder during the last week on a scale from 0 to 4. A zero indicates that the patient did not experience the item and four indicates the most severe reaction. This self-report scale has good internal reliability, test-retest reliability and sensitivity to change [70]. The **Mobility Inventory for Agoraphobia** (MIA) [71] assesses the frequency of agoraphobic avoidance behaviour by asking patients to evaluate their level of avoidance of 27 places or situations when alone or when with a companion. Each item is given a rating from 1 to 5 where 1 represents no avoidance and a 5 means that the place or situation is always avoided. The MIA has been proven to have excellent internal consistency, and strong convergent and discriminant validity [71]. The **Patient Health Questionnaire** (PHQ-9) [72] is used for the assessment of the frequency of depressive symptoms with good reliability and validity. Patients indicate how many days in the last 2 weeks they have experienced any of 9 symptoms of depression with responses ranging from 0, meaning never, to 3 meaning almost every day. Scores below 10 indicate unlikely major depression while scores above 15 indicate likely major depression. The **Insomnia Severity Index** [73, 74] is a brief 7-item questionnaire assessing the severity of insomnia and associated difficulties. Each item is rated on a 0 to 4 scale, total score ranging from 0 to 28, with a higher score suggesting more severe insomnia. The instrument has been shown to have adequate internal consistency, concurrent validity documented by significant correlations with sleep diary and polysomnography measures. The **Sheehan Disability Scale** [75] is a measure that allows patients to indicate their level of disability visually, numerically, or descriptively on a scale for each of 3 life dimensions: work, social life and family life. Each item is scored from 0 being no impairment, to a 10 being extremely impaired. It is a brief rating scale that has good sensitivity and a score of 5 or higher corresponds with an increased risk of psychiatric impairment. The **EuroQoL EQ-5D** [76] is a 5-item standardised scale measuring health-related quality of life on five dimensions (e.g., mobility, self-care, anxiety/depression), each rated on 5 levels of impairment. The instrument includes a visual analogue scale to rate health from 0 to 100. The scale has excellent test-retest reliability; and good concurrent validity with the SF-36. The **Centers for Disease Control Healthy Days Measures** (CDC HRQOL-4) [77] includes one item of the perception of one’s health and three items assessing the number of days in the past 30 days when physical or mental health was not good and that activity limitations were present. The scale has demonstrated acceptable test-retest reliability and strong internal validity. The list of 21 chronic diseases from the **Disease Burden Morbidity Assessment** [78, 79] was included to document the presence of chronic conditions. The ratings of the interference of conditions on daily activities were not included. The **Mental Health Continuum Short Form** (MHC-SF) [80] consists of 14 items measuring the frequency at which respondents experience emotional, psychological, and social well-being in the past month. Items are rated from 0 (Never) to 5 (Every day). The short form of the MHC has shown excellent internal consistency, construct and discriminant validity. The **Mental Health Self-Management Questionnaire** (MHSQ) [81] assesses the use of mental health self-management strategies. It comprises 18 items rated on a 5-point Likert scale ranging from 0 (Never) to 5 (Very often), with a total maximum score of 72. The scale has satisfactory internal reliability and construct validity, adequate test–retest reliability and its convergent and concurrent validity are supported. The **Medical Outcomes Study Social Support Survey** (MOS-SSS) [82, 83] is a 6-item version of the original MOS scale which assesses the types of support and their availability on a scale from 1 (None of the time) to 5 (All of the time). The instrument has strong internal consistency and scale reliability.

#### Questionnaires completed during therapy sessions

At the onset of each session, participants complete the **Anxiety Disorder Diagnostic Questionnaire - weekly** (ADDQ-W) [84], a version adapted from the original ADDQ [85] for session-to-session transdiagnostic assessment of change in clinical fear and anxiety. The ADDQ-W possesses strong internal consistency. After sessions 3 and 9, the participants and therapists complete the 12-item versions of the **Working Alliance Inventory (WAI)** to assess the quality of the therapeutic relationship [86, 87]. The two short-form versions of the WAI include 12 items rated on a scale ranging from 1 (Never) to 7 (Always), a greater score reflecting a better alliance. The WAI is widely used in psychotherapy, and possess established construct validity and high internal consistency [88]. After sessions 1 and 12, participants complete the 6-item **Credibility Expectancy Questionnaire** [89] which assesses their perceptions of the improvement they think and feel will occur (session 1) and that did occur (session 12). The CEQ possesses high test-retest reliability high internal consistency. After the ninth session, participants complete the **Gross Cohesion Scale** (GCS) [90], a 9-item scale evaluating the perceived cohesiveness and bond among group members. The scale has acceptable reliability and validity.

For each participant, the therapists record the following information after each session: presence, homework completion, participation level, and clinical observations. The logbook content provides a better understanding of therapists’ experience, add to the evaluation of therapeutic integrity, and contribute to the optimization of the program for future large-scale implementation of tCBT. The therapists also complete a brief questionnaire comprising sociodemographic questions, items on academic and professional backgrounds, as well as experience with CBT, group therapy and treatment of anxiety disorders.

#### Linkages with administrative data

Health care utilization data will be obtained retrospectively from provincial administrative databases (e.g., computerized database of the *Régie de l’assurance-maladie du Québec* (RAMQ) for medical services, hospitalization’s registry, and medication data) after the 12 month follow-up (T_4_).

#### Sample size

It was not feasible to estimate the sample size required for a mixed regression model as current guidelines are only generalizable to particular data conditions and model structures [91]. Therefore, as an alternative, we calculated sample size for a univariate comparison for each of the primary outcomes (symptoms of anxiety as measured with the BAI and the ADIS-5) between the two study groups at post-treatment. The sample size calculation is based on a conservative estimated effect size of the intervention (Cohen’s *d*) of 0.45 at T_1_, according to previous studies [28, 34, 45, 64, 92, 93]. From pilot trial data, we estimated the correlation between the two primary outcomes to be approximately 0.7. To minimize the type-1 error associated with multiple primary endpoints, the common alpha value of 5% was adjusted to 0.03 [94]. A sample size of 182 individuals will therefore be required to detect a minimal important difference between the CBT and TAU groups, assuming an 80% power, and two-tail tests. The sample size was calculated using G*Power.

With an estimated 15% rate of loss at follow up factored in, the proposed final sample size is thus 215 patients in total (107 and 108 for each arm respectively). Such sample size will give us sufficient power to detect differences in the primary outcomes from a longitudinal perspective.

#### Recruitment

**Fig. 1:**
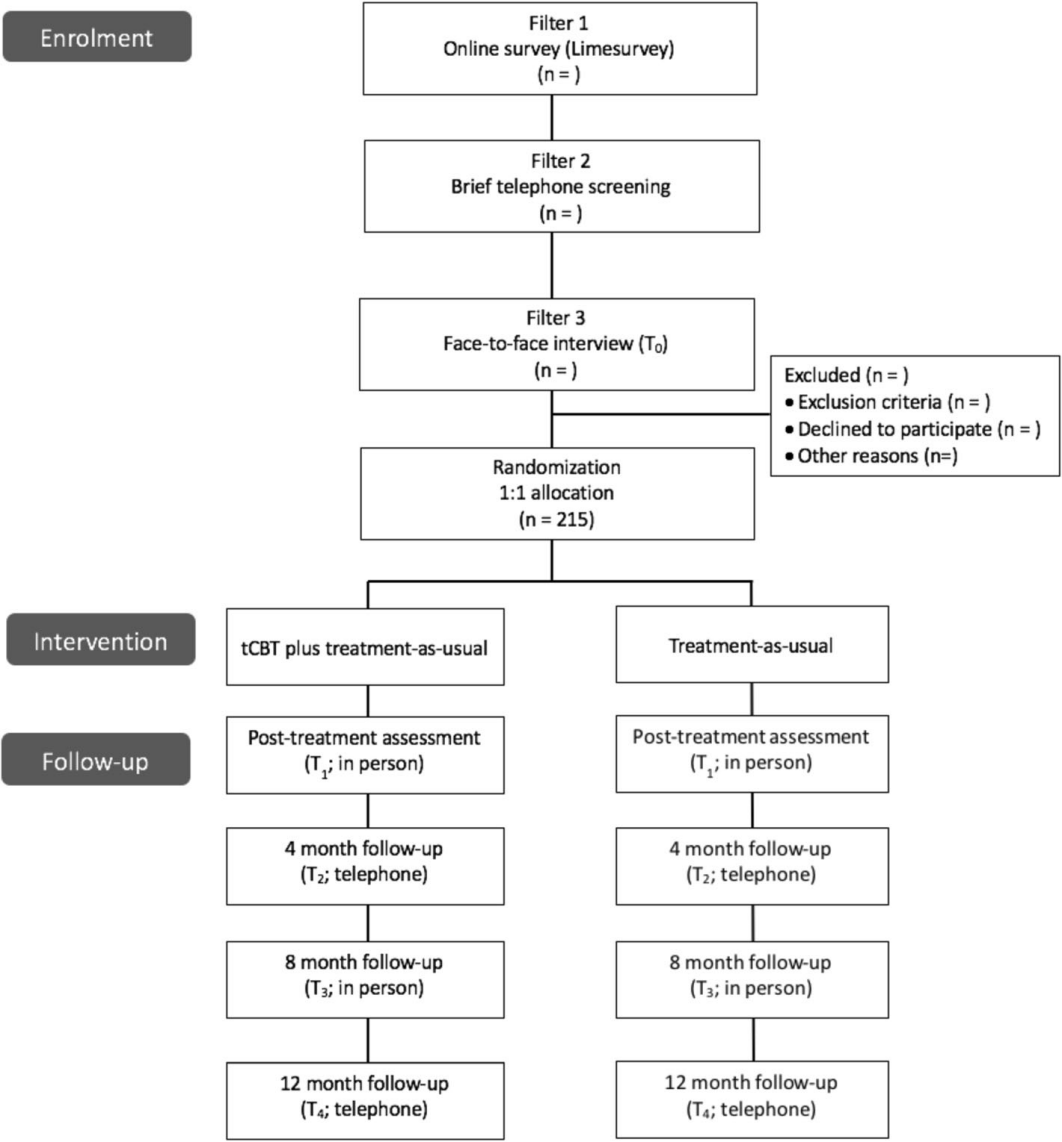
Flow of participants

As shown in Fig. 1, our pragmatic recruitment approach is conducted through a three-stage process and aims at recruiting a range of participants that would typically seek care in the primary care setting for anxiety disorders, such as new cases of anxiety disorders, longstanding sub-optimally treated patients, patients with psychiatric or physical comorbidity, patients with previous or ongoing treatment experience, etc.

Filter 1: We advertise the study in the general population and primary care settings through the following strategies: 1- publicity in regional newspapers; 2- targeted ads to geographic locations with Facebook and Google AdWords; 3- advertisement posters on bulletin boards of medical clinics and pharmacies, via targeted mass mail-out including a letter from the research team and a recruitment poster; 4- convenience advertisement from the research team in diverse public locations. Self-referred individuals then acquire further information on the study by consulting the study’s website or through a telephone call or email to research coordinator (AB). Self-referred individuals have to complete a brief online survey with LimeSurvey that they access through the study’s website. The screening survey contains basic eligibility criteria as well as anxiety symptoms and comorbidity overview. The survey includes the **Generalised Anxiety Disorder-7** (GAD-7) [95], a brief screening tool assessing the frequency of the 7 items in the past 2 weeks. Each item is scored from 0 (Not at all) to 3 (Nearly every day), providing a 0 to 21 severity score. To confirm that anxiety symptomatology is present, a cut point value ≥8 is used. The GAD-7 has good reliability as well as strong criterion validity. Initially designed for GAD detection, this instrument has high sensitivity and good specificity for detecting generalized anxiety disorder and other anxiety disorders [11, 96]. The survey also includes the 2-item **Patient Health Questionnaire** (PHQ-2) [97], a very brief depression screener comprising the first 2 items of the PHQ-9 [72]. The items assess the frequency of depressed mood in the past 2 weeks and are scored from 0 (Not at all) to 3 (Nearly every day), for a total severity score from 0 to 6. The instrument’s construct and criterion validity have been established. The presence of possible alcohol abuse and dependence is assessed with the **CAGE** (Cut-down, Annoyed, Guilty, Eye-opener), a widely used screening instrument [98, 99]. It comprises four items scored 0 (No) or 1 (Yes). The total score ranges from 0 to 4, with a recommended cut-off of ≥2 to screen for alcohol abuse. The CAGE has a high test-retest reliability, and adequate validity for detecting alcohol abuse and dependence in various patient populations [100]. General 1 or 2-item screening questions explore the presence of symptoms associated with Panic Disorder, Agoraphobia, Social Anxiety Disorder, Generalized Anxiety Disorder, Obsessive-Compulsive Disorder and Posttraumatic Stress Disorder in the past 6 months. Participants are asked if a health professional had already diagnosed an anxiety or mood disorders, and if they accessed resources for their anxiety in the past 2 years (support group, individual or group therapy). At the end of the survey, they provide their name and contact information. In the presence of clear exclusion criteria at the end of the survey, a list of mental health resources is provided. Filter 2: In the second stage, clinical evaluators contact individuals meeting basic eligibility criteria for a brief telephone-screening interview based on a semistructured questionnaire and clinical profile from their LimeSurvey answers. Filter 3: In the third stage, face-to-face assessment interviews are conducted with potential candidates to assess their eligibility based on the ADIS-5 assessment (T_0_).

#### Participant timeline

Data collection is managed independently of the treatment assignment by interviewers who are blind to the participant’s treatment assignment. The initial assessment (T_0_; random pre-assignment) is conducted in person and comprises completion of the consent form, the administration of the ADIS-5 and the questionnaire on sociodemographic data, health care costs, medication and work performance by a trained PhD level evaluator. Patients meeting eligibility criteria are given a participant’s booklet with the self-reported questionnaires and a stamped envelope to return the questionnaires to the research team within 48 h, and only then do we have all required information to proceed with randomization. The same booklet is being used at T_1_, T_2_, T_3_ and T_4_. The follow-up assessments take place at post-treatment (T_1;_ in person), 4 months (T_2_; service utilization only; telephone), 8 months (T_3_; in person) and 12 months (T_4_; service utilization only; telephone) follow-up.

Strategies to minimize loss to follow up include multiple study design and site personal approaches. We minimize participants’ burden and inconvenience through a minimal number of data collection, user-friendly patient booklets, secondary direct data capture through provincial administrative databases, and convenient location in a primary care or university setting. To enhance personal contact with participants, a considerate research coordinator is the primary contact throughout the trial by telephone or email. We recruited experienced interviewers and provided training for the minimization of missing data. Financial incentives ($20 CAD) are offered to participants for each in-person assessment. The group tCBT intervention is also offered to participants in the TAU group at the 12-month follow up to increase perceived health benefits associated with the trial.

### Assignment of interventions: Sequence generation, allocation concealment mechanism and implementation

Randomization is at the patient level. Contamination is unlikely because specific CBT treatments for anxiety disorders are rarely available in primary care in our health care context. To ensure that comparable groups are obtained, stratification based on site is implemented. To ensure a balance in the allocation for the strata and thus eliminate the risk of a secular trend in the composition of groups, blocks of four are used. This strategy also helps to avoid differences in the composition of the two groups, in the event of changes in clinical characteristics of those involved in the study over time. Randomization is being carried out using a code generated by a statistical software (manages the stratification and blocks) with a ratio of 1:1, set up by an independent statistician who is not be involved in the recruitment.

Allocation concealment is ensured by an online computer tool for clinical trials (PIERCE) that only releases the randomization code to the principal investigator (PR) after the baseline assessment is completed. Upon review of data entry on the PIERCE computer tool from research coordinator and clinical evaluators concerning inclusion and exclusion criteria, written informed consent, agreement to accept the randomly assigned treatment and returned participant’s questionnaire booklet, a computer command allows release of the randomization code (PR). Each member of the team (research coordinator, clinical evaluators, principal investigator) has his or her own access code to the PIERCE system, designed with several access layers, and the randomization module is only accessible to the principal investigator (PR). She informs the research coordinator of each treatment assignment based on the allocation sequence, and the treatment allocation is be communicated to participants by telephone within 2 weeks after the initial clinical interview and reception of the booklet.

#### Blinding

Blinding of trial participants and therapists is not possible in this trial. Concealment of the treatment allocation is maintained for the clinical evaluators, research team and data analyst, with the exception of the research coordinator. Participants are asked not to discuss group participation with clinical evaluators. The randomization sequence will not be broken in the data set, with random codes (“A” and “B”), until the analyses for the primary outcomes are completed. The sequence for a specific participant will be broken in case of an incident if requested by the independent Data Safety and Monitoring Committee (DSMC).

### Data collection, management, and analysis

The clinical assessment of participants is conducted in person (T_0_, T_1_, T_3_) by trained PhD level evaluators at the three sites or by telephone (T_2_ and T_4_; interview on health care costs) by the research coordinator (AB). The evaluators received a one-day training on the ADIS-5 as well as additional training to ensure optimal data collection (e.g., missing data, patient retention). Regular supervision (MDP, PG) is provided to clinical evaluators throughout the duration of the data collection, particularly regarding the ADIS-5 assessments. For the post-treatment and follow-up assessments, participants also receive a letter by mail reminding them of the upcoming clinical evaluation, including a booklet with a stamped envelope. The research coordinator manages contacts with participants for scheduling assessments.

A procedure is followed to promote participant retention and complete follow-up. Treatment and follow-up are managed as different functions by different research team members, with the data collection schedule independent of treatment status and adherence to protocol. The T_2_ telephone assessment of participants contributes to avoid recall bias for service utilization as well as maintain patient’s interest in the study by regular contact with research personnel in between face-to-face assessment periods. In particular cases of participants from either groups non-compliant with follow-up assessment periods, we try to minimize the assessment burden by having them complete only the BAI primary outcome rather than the full evaluation.

Data entry, coding, storage and analysis is managed (PR) at Université de Sherbrooke on a secure server with systematic backups. Double data entry is being conducted for 10% of patient questionnaires throughout the trial to promote data quality, and data entry is under the responsibility of a trained research assistant.

#### Control for bias sources

The primary means to avoid bias include the randomization scheme; the masked data collection by clinical evaluators who enrol and evaluate participants throughout the project while being uninformed of treatment assignment (with an incident report and reassignment procedure if they become unmasked); the masked research hypotheses for participants.

#### Statistical analyses for primary outcome measures

##### Clinical outcomes

Statistical analysis of the data will follow intention-to-treat principles [101], i.e. “1) keep participants in the intervention group to which they were randomized, regardless of the intervention they actually received; 2) measure outcome data on all participants; 3) include all randomized participants in the analysis.” Baseline sociodemographic and clinical status (type of anxiety disorder, comorbidity, symptom severity, functional status, current treatment) will be described by intervention group. A mixed effects regression model will be used to account for between- and within-subject variations in the longitudinal effects of the intervention on the primary outcome measures of anxiety. Such model will allow for the inclusion of patients with missing data at any of the follow-up interviews as well as the within effect of intervention (maintenance of gains within individuals). The effects of treatment will also be adjusted for three covariates: a) comorbid depressive symptoms (continuous; PHQ-9 score); b) psychotropic medication (yes or no); c) specific anxiety disorder (ADIS-5). This set of variables will be added to the mixed regression model described above to better understand the additive contribution of each variable to the relationship between the study groups and anxiety. Functioning and quality of life outcomes also measured longitudinally will be analyzed in a similar fashion (a generalized estimating equation (GEE) with a logit-link function regression model will be used when necessary). Sub-group analyses for the presence of an antidepressant and/or benzodiazepines medication, the principal anxiety disorder at baseline and gender are planned. Treatment effect sizes will be calculated with Cohen’s d for each sub-group of interest. Compliance rates (number of sessions completed) will be compared using t-tests. Additionally, sensitivity analysis [102] will be conducted to assess the impact of missing data on estimates of treatment effects with an adequate multiple imputation statistical technique [102] and without imputation (i.e. available case analyses), as well as for outlying observations and “per protocol”. Reporting of sensitivity analysis will provide valuable information on the robustness of results.

## Discussion

Our pragmatic clinical trial will deliver important data to patients, clinicians, health care managers and decision makers to inform the implementation of optimal mental health services in real-world practice with clinically relevant outcomes. There is a major gap between knowledge and practice with regards to evidence-based psychotherapy for anxiety disorders, and a dissemination priority should be CBT, for which there is a substantial evidence base. While numerous studies have examined quality improvement approaches for the primary care management of depression, anxiety disorders have received limited attention. tCBT is a promising intervention for the large-scale implementation of CBT to improve access to evidence-based psychological treatments for patients with mixed anxiety disorders. In this pragmatic trial, we aim at examining the relative effectiveness of tCBT as a complement to usual care in the real world from a primary mental health care standpoint to generate evidence useful for decision-makers in the implementation of change in routine clinical practice for anxiety disorders. Currently, there is positive momentum to influence the organization of services given the resources invested in strengthening primary mental health care and the imperative to improve access to psychotherapy. A pragmatic trial of group tCBT will help to guide decision makers, managers and clinicians who are entrusted to develop mental health policies and implement mental health services. Among strengths of our pragmatic trial, the study will be rooted in the current reality of primary mental health care with a large sample of patients characteristic of primary care, for instance with multiple anxiety disorders, comorbid depression or concomitant pharmacological treatments. We will balance fidelity and pragmatism of treatment delivery with co-therapists that come from primary mental health care as well as CBT experts. The choice of usual care as a comparator will provide a better estimate of CBT effect size than a waiting-list condition [28], with no restrictions on other ongoing or new treatments. Among study limitations, current diagnosis-specific and global anxiety assessment tools certainly present limitations for the transdiagnostic assessment of multiple anxiety disorders [84]. We tried to palliate this limitation by selecting the widely used self-reported BAI for comparability and the clinician-rated ADIS-5 for the principal disorder, and diagnosis-specific secondary outcome measures will provide essential complementary data on outcomes. Satisfactory results of this trial could lead to a significant improvement in access to evidence-based psychotherapy for anxiety disorders in primary mental health care settings.

### Trial status

Patient enrolment began on September 12th 2016 and was completed February 19th 2018. The final treatment group is planned for completion in May 2018.

## Abbreviations

ADDQ-W: Anxiety Disorder Diagnostic Questionnaire - weekly
ADIS-5: Anxiety and Related Disorders Interview Schedule for DSM-5
BAI: Beck Anxiety Inventory
CBT: Cognitive Behaviour Therapy
CDC HRQOL-4: Centers for Disease Control Healthy Days Measures
CSR: Clinical Severity Rating
GCS: Gross Cohesion Scale
HPQ: World Health Organization Health and Work Performance Questionnaire
MHC-SF: Mental Health Continuum Short Form
MHSQ: Mental Health Self-Management Questionnaire
MIA: Mobility Inventory for Agoraphobia
MOS-SSS: Medical Outcomes Study Social Support Survey
PDSS-SR: Panic Disorder Severity Scale Self Report
PHQ-9: Patient Health Questionnaire
PSWQ: Penn State Worry Questionnaire
SPIN: Social Phobia Inventory
TAU: Treatment-as-usual
tCBT: Transdiagnostic CBT
WAI: Working Alliance Inventory
